# A Phase II Study of Docetaxel for the Treatment of Recurrent Osteosarcoma

**DOI:** 10.1080/13577140410001711764

**Published:** 2004

**Authors:** Anne Mctiernan, Jeremy S. Whelan

**Affiliations:** Meyerstein Institute of Oncology Middlesex Hospital UCL Hospitals NHS Trust Mortimer St London W1T 3AA UK

## Abstract

*Purpose*: To determine the response and toxicity of docetaxel in recurrent osteosarcoma and related spindle cell tumours of bone.

*Patients and methods*: Fourteen patients, 10 males and four females, were enrolled, median age 30.5 years (range, 17–46). Diagnosis was: conventional osteosarcoma, 12 patients; periosteal osteosarcoma, one patient; and malignant fibrous histiocytoma of bone, one patient. Initial chemotherapy had been with doxorubicin and cisplatin in 10 patients, and multiagent regimens in four. Nine had been treated with second line chemotherapy before receiving docetaxel. Thirteen patients had lung metastases and one intra-abdominal disease. Docetaxel 100 mg/mα^2^ was given as a 1-h infusion every 3 weeks. Response was assessed every two cycles to a maximum of six.

*Results*: A total of 43 cycles were given, median of two per patient (range 1–6). Thirteen patients were evaluable for response. A single partial remission was seen, for a response rate of 8%. Two patients had stable disease, and one patient a mixed response. Forty cycles were evaluable for toxicity. The principle toxicity was haematological, with a median neutrophil count of 0.9 (range 0–9.6). There were four episodes of neutropenic sepsis (10%). The only non-haematological toxicity ≥grade 3 was stomatitis, occurring in just one patient. There were no toxic deaths.

*Conclusion*: Docetaxel at this dose and schedule is well tolerated, but is not associated with significant activity in patients with relapsed osteosarcoma.


*Sarcoma*, June/September 2004, *VOL. 8, NO. 2/3, 71–76*

Taylor&Francis healthsciences

**ORIGINAL ARTICLE**

### A Phase II study of docetaxel for the treatment of recurrent osteosarcoma

ANNE McTIERNAN & JEREMY S. WHELAN

*Meyerstein Institute of Oncology, Middlesex Hospital, UCL Hospitals NHS Trust, London, UK*

**Abstract**

*Purpose*: To determine the response and toxicity of docetaxel in recurrent osteosarcoma and related spindle cell tumours of bone.

*Patients and methods*: Fourteen patients, 10 males and four females, were enrolled, median age 30.5 years (range, 17–46). Diagnosis was: conventional osteosarcoma, 12 patients; periosteal osteosarcoma, one patient; and malignant fibrous histiocytoma of bone, one patient. Initial chemotherapy had been with doxorubicin and cisplatin in 10 patients, and multiagent regimens in four. Nine had been treated with second line chemotherapy before receiving docetaxel. Thirteen patients had lung metastases and one intra-abdominal disease. Docetaxel 100 mg/m$^2$ was given as a 1-h infusion every 3 weeks. Response was assessed every two cycles to a maximum of six.

*Results*: A total of 43 cycles were given, median of two per patient (range 1–6). Thirteen patients were evaluable for response. A single partial remission was seen, for a response rate of 8%. Two patients had stable disease, and one patient a mixed response. Forty cycles were evaluable for toxicity. The principle toxicity was haematological, with a median neutrophil count of 0.9 (range 0–9.6). There were four episodes of neutropenic sepsis (10%). The only non-haematological toxicity $\geq$ grade 3 was stomatitis, occurring in just one patient. There were no toxic deaths.

*Conclusion*: Docetaxel at this dose and schedule is well tolerated, but is not associated with significant activity in patients with relapsed osteosarcoma.

**Key words:** *Taxotere, taxanes, osteosarcoma, bone tumours*

### Introduction

The outlook of patients with osteosarcoma has dramatically improved over the last 25 years, so that with the use of multi-agent chemotherapy and surgery almost two-thirds of patients can expect to be cured.[1–5]

However, for patients who present with unresectable or relapsed disease the outlook remains poor.[6,7] For those with recurrent disease, surgery is the most valuable treatment modality if cure is to be achieved, but in some studies second line chemotherapy has also been associated with improved outcome.[7–9] In order to improve the outlook for this group of patients, and achieve further improvements in those with good prognosis disease, new agents, active against this disease, are needed.

Docetaxel is a semi-synthetic taxane, which in common with other taxanes, promotes microtubule assembly and inhibits disassembly thereby causing cellular growth arrest.[10] Activity to docetaxel has been identified in a wide range of tumours including ovarian cancer,[11,12] breast cancer,[13–15] gastric cancer,[16] non-small lung cancer[17] and limited activity in some sarcomas.[18–21] Sensitivity to docetaxel has also been demonstrated in different osteosarcoma cell lines,[22,23] although resistance was shown to develop in one of these studies.[23]

Synergistic activity of the combination of docetaxel and cisplatin or carboplatin has been demonstrated in various tumours.[24–26] As cisplatin is one of the active agents in the treatment of osteosarcoma, the identification of synergy with the taxoids could be of potential significance, if response to docetaxel in osteosarcoma was shown.

The aim of this study was to determine the activity of docetaxel in patients with relapsed or refractory osteosarcoma and related spindle cell sarcomas of bone.

Correspondence to: J.S. Whelan, Meyerstein Institute of Oncology, Middlesex Hospital, UCL Hospitals NHS Trust, Mortimer St, London W1T 3AA, UK. Tel.: +44-207-380-9346; Fax: +44-207-436-0160; E-mail: jeremy.whelan@uclh.org

1357-714X print/1369–1643 online © 2004 Taylor & Francis Ltd
DOI: 10.1080/13577140410001711764

### Patients and methods

### *Eligibility*

Patients aged between 14 and 70 years with relapsed or refractory histiologically proven osteosarcoma or malignant fibrous histiocytoma of bone (MFH-B) were eligible for this study. Eligibility criteria included: WHO performance status $\leq 3$ with a life expectancy of greater than 8 weeks; measurable or assessable disease; and adequate organ function, defined as neutrophils $\geq 1.5 \times 10^9$/l; platelets $\geq 100 \times 10^9$/l; serum creatinine $\leq 1.5 \times$ upper normal limit (UNL); bilirubin $\leq 1 \times$ UNL; AST and/or ALT $\times$ $1.5 \times$ UNL; and alkaline phosphatase $\leq 2.5 \times$ UNL (unless bone metastases present in the absence of any liver disorder). Exclusion criteria included co-existing illness precluding chemotherapy; pregnant or lactating women; symptomatic peripheral neuropathy $\geq$grade 2; history of severe hypersensitivity to polysorbate 80; and contraindications to the use of steroids. The protocol was reviewed and approved by a local ethics committee, and written informed consent was obtained from patients and/or parents where appropriate.

### *Pre-treatment evaluation*

At study entry patients had a complete history and physical examination, including performance status, assessment of residual toxicity and clinical tumour measurements. Blood tests including full blood count, chemistry, baseline alkaline phosphatase and lactate dehydrogenase were also performed. Imaging included chest X-ray and computed tomography (CT) of the chest, isotopic bone scan, and plain X-rays or magnetic resonance imaging (MRI) of the primary tumour where appropriate.

### *Treatment*

Docetaxel 100 mg/m$^2$ was given on an outpatient basis, as a 1-h intravenous infusion every 21 days, for a maximum of six cycles. Dexamethasone, 8 mg twice daily, was given as a pre-medication, starting the day before the infusion, and continued for 5 days in total.

Patients were clinically reassessed every 3 weeks, including clinical history since previous infusion and assessment of toxicity. Full blood count and chemistry was undertaken before the start of each cycle, and full blood tests also performed at days 8 and 15 of each cycle. In the event of continuing toxicity, treatment was delayed for a maximum of 2 weeks to allow for haematological recovery of neutrophils to $\geq 1.0$ or platelets $\geq 75$, or of non-haematological toxicity to grade 1 or below. In the event of prolonged neutropenia (neutrophils $<0.5$ for more than 7 days) or neutropenic sepsis, the dose of subsequent cycles was reduced by 25%. In the event of grade 4 neutropenia of $\leq 7$ days without neutropenic sepsis, granulocyte-colony stimulating factor (G-CSF) was considered.

### *Evaluation of response*

Response to docetaxel was assessed after every two cycles of chemotherapy according to the WHO criteria for clinical response.[27] Complete response (CR) was defined as the total disappearance if all lesions determined by two observations, not less than 4 weeks apart; partial response (PR) was the decrease in the sum of at least 50% in the sum of the products of the largest perpendicular dimensions of all measurable lesions; a minor response (mR) indicated the decrease of $\geq 25\%$, but $<50\%$ in the sum of all measurable lesions; and progressive disease (PD) indicated an increase of $\geq 25\%$ in the sum of the largest perpendicular dimensions of all measurable lesions or the appearance of new disease at any site. Stable disease (SD) was less than 25% decrease or increase in all measurable lesions, and the absence of any new disease.

Toxicity was graded according to the National Cancer Institute – Common Toxicity Criteria (NCI-CTC), version 2.0.[28]

### Results

The patient characteristics are shown in Table 1. A total of 14 patients, 10 males and four females were entered. The median age at study entry was 30.5 years (range 17–46). Diagnosis was: conventional osteosarcoma in 12 patients; periosteal osteosarcoma in one patient; and MFH-B in one. Eleven patients (79%) had presented with localised extremity tumours at diagnosis, two with extremity tumours with lung metastases, and one patient with a localised pelvic tumour. First line chemotherapy had been with doxorubicin and cisplatin in 10 patients, and multiagent regimens in the remaining four. All had undergone surgery to the primary, three with adjuvant radiotherapy.

The timing of treatment with docetaxel was first recurrence in four patients, and second or subsequent recurrence in 10. Nine patients had received prior chemotherapy for relapsed disease, seven with surgery. One further patient had undergone surgery and adjuvant radiotherapy for a local recurrence 7 months prior to treatment with docetaxel for a metastatic recurrence in the lung. The median number of previous chemotherapy regimens received was 2 (range, 1–3) and the median number of previous chemotherapy agents received was 4 (range, 2–9).

Site of disease at the start of treatment with docetaxel was: lung in eight patients, combined local and lung metastases in two, lung and bone in two, lung and subcutaneous metastases in one, and intra-abdominal disease in one. The median

**Table 1.** *Patient characteristics and response*

| Patient | Diagnosis | Age (years) | Primary | First line chemotherapy | Local therapy | Prior chemotherapy for relapse | Timing of docetaxel | Site of disease | Docetaxel | |
|---|---|---|---|---|---|---|---|---|---|---|
| | | | | | | | | | No. of cycles | Best response |
| 1 | OS | 46 | Distal femur | Dox + Cisp × 6 | EPR | None | $1^{st}$ Recurrence | Lung | 2 | PD |
| 2 | OS | 30 | Distal femur | Multi agent | EPR | Ifo + Eto × 6 | $>2^{nd}$ Recurrence | Lung & Bone | 2 | PD |
| 3 | MFH-B | 44 | Proximal humerus | Dox + Cisp × 5 | EPR | Ifo + Eto × 6; HD-Mtx | $>2^{nd}$ Recurrence | Lung | 2 | PD |
| 4 | OS | 17 | Proximal tibia | Dox + Cisp × 5 | EPR | None | $2^{nd}$ Recurrence | Local & Lung | 6 | SD |
| 5 | OS | 19 | Proximal tibia | Dox + Cisp × 6 | EPR + RT | HD-Mtx; Ifo + Eto × 5 | $>2^{nd}$ Recurrence | Lung | 2 | PD |
| 6 | OS | 33 | Pelvis | Dox,Cisp + Ifo × 6 | Hemi-pelvectomy + RT | None | $1^{st}$ Recurrence | Hypochondrium | 1 | PD |
| 7 | OS | 23 | Proximal tibia | Dox + Cisp × 4 | EPR | Ifo + Eto × 2 | $>2^{nd}$ Recurrence | Local & Lung | 1 | (SD)[a] |
| 8 | OS | 42 | Proximal tibia | Dox + Cisp × 3 HD-Mtx × 1; Dox + Carb × 2 | Amputation | Ifo + Eto × 6 | $>2^{nd}$ Recurrence | Lung | 2 | PD |
| 9 | Per-OS | 33 | Distal femur | Dox + Cisp × 4 | EPR | Dox + Cisp × 2; Ifo+Eto × 6 | $>2^{nd}$ Recurrence | Lung & Bone | 4 | PD |
| 10 | OS | 33 | Proximal humerus | Dox + Cisp × 6 | EPR + RT | None | $1^{st}$ Recurrence | Lung | 2 | PD |
| 11 | OS | 21 | Proximal humerus | Dox + Cisp × 4; Ifo × 2 | EPR | None | $1^{st}$ Recurrence | Lung | 6 | SD |
| 12 | OS | 17 | Proximal tibia | Dox + Cisp × 6 | Amputation | Ifo + Eto × 4; HD-Mtx × 3 | $>2^{nd}$ Recurrence | Lung | 6 | PR |
| 13 | OS | 31 | Proximal humerus | Dox + Cisp × 6 | EPR | Ifo + Eto × 6 | $>2^{nd}$ Recurrence | Lung + Subcutaneous nodule | 5 | PD (Mixed) |
| 14 | OS | 19 | Proximal tibia | Dox + Cisp × 6 | EPR | Ifo + Eto × 6; HD-Mtx × 6 | $>2^{nd}$ Recurrence | Lung | 2 | PD |

[a]Not formally evaluable for response as changed to carboplatin following anaphylactic reaction to docetaxel

Abbreviations: OS, conventional osteosarcoma; Per-OS, periosteal osteosarcoma; MFH-B, malignant fibrous histiocytoma of bone; Dox, doxorubicin; Cisp, cisplatin; Carb, carboplatin; Ifo, ifosfamide; HD-Mtx, methotrexate 12 g/m$^2$; Eto, etoposide; EPR, endo-prosthetic replacement; RT, radiotherapy; PD, progressive disease; SD, stable disease; PR, partial response.

**Table 2.** *Treatment-related toxicity*

| Grade | Cycles with toxicity No. (%) 1 | 2 | 3 | 4 | Patients with toxicity (all grades) No. (%) |
|---|---|---|---|---|---|
| *Toxicity* | | | | | |
| White blood count | 11 (28) | 12 (30) | 3 (8) | 5 (13) | 13 (93) |
| Neutropenia | 4 (10) | 3 (8) | 11 (28) | 11 (28) | 13 (93) |
| Anaemia | 29 (73) | 7 (18) | 2 (5) | | 13 (93) |
| Thrombocytopenia | 1 (3) | | | 2 (5) | 3 (21) |
| Anaphylaxis | | 1 (3) | | | 1 (7) |
| Arthralgia | 1 (3) | 1 (3) | | | 1 (7) |
| Lethargy | 5 (13) | 4 (10) | | | 8 (57) |
| Myalgia | 3 (8) | | | | 3 (21) |
| Diarrhoea | 6 (15) | 1 (3) | | | 6 (43) |
| Nausea | 4 (10) | 2 (5) | | | 3 (21) |
| Vomiting | | 1 (3) | | | 1 (7) |
| Stomatitis | 10 (25) | 8 (20) | 2 (5) | | 9 (64) |
| Infection | | 1(3) | 4 (10) | | 5 (36) |
| Constipation | 3 (8) | 3 (8) | | | 4 (29) |
| Headache | | 1 (3) | | | 1 (7) |
| Neuropathy | 3 (8) | 1 (3) | | | 2 (14) |

Number of evaluable cycles = 40; total number of patients = 14.

treatment-free interval before the start of docetaxel was 44 weeks (range, 14–454).

A total of 43 cycles were given, with a median of two per patient (range, 1–6). Dose reductions were required in eight of the 43 cycles (19%), affecting five patients. Dose reductions were given for neutropenic sepsis in four patients, and cumulative toxicity in one. Later in the series, two heavily pre-treated patients received prophylactic G-CSF from the start of treatment. Cycles were given at a median of every 21 days, with only one cycle being delayed to coincide with a clinic visit.

Forty cycles were assessable for toxicity (Table 2). The principle toxicity was haematological, with a median neutrophil count of 0.9 (range, 0–9.6). Four cycles (10%) were complicated by neutropenic sepsis. Only one patient experienced a non-haematological toxicity of greater than grade 2, developing a grade 3 stomatitis after two consecutive cycles of chemotherapy. However, one patient experienced an immediate anaphylactic reaction (grade 2) to his second cycle of docetaxel, which resolved spontaneously when the infusion was stopped. As there had been no response observed after the first cycle of docetaxel, no further docetaxel was given and the patient elected to receive single-agent carboplatin. Other grade 1–2 non-haematological toxicities included: stomatitis in eight patients; lethargy in eight patients; diarrhoea in six patients; constipation in four patients; myalgia in three patients; nausea in three patients; neuropathy in two patients; and vomiting, arthralgia, and headache in one patient each.

### *Response*

Thirteen patients were evaluable for response. The remaining patient switched from docetaxel to carboplatin after an anaphylactic reaction to docetaxel, but had stable disease after the first cycle. Of the 13 patients who remained evaluable for response, only one PR was observed, lasting 9 weeks, for an overall response rate of 8%. Two patients had stable disease for durations of 15 and 33 weeks, respectively. One further patient had a mixed response with a greater than 50% reduction of a cutaneous metastasis and reduction in pre-existing lung metastases, but developed synchronous metastases during treatment, giving an overall response of PD. All 14 patients have subsequently died of progressive disease, at a median of 8 months from the start of docetaxel chemotherapy (range, 1–20 months).

### Discussion

The prognosis of patients with localised extremity osteosarcoma has improved dramatically with the use of multi-agent chemotherapy in addition to surgery over the past few decades. However, for those with unresectable or recurrent disease the prognosis remains poor. The response rate of just 8% to docetaxel in this study is therefore disappointing, suggesting little activity at this dose and schedule in patients who relapse with osteosarcoma after conventional chemotherapy. Furthermore the only response seen was of a very short duration (9 weeks).

There are few published data examining the efficacy of docetaxel in patients with osteosarcoma. In a phase I dose-escalating study of docetaxel 55–150 mg/m$^2$ in paediatric solid tumours, no responses were observed in 11 patients with osteosarcoma.[29] Similarly, in a subsequent dose-escalating study of docetaxel 150–235 mg/m$^2$ with filgrastim support in paediatric patients, no responses were seen in a further nine patients with osteosarcoma.[30]

Another taxane which has been studied in osteosarcoma is paclitaxel. Patel *et al.*[31] treated 15 patients with osteosarcoma and its variants (i.e., including three patients with MFH-B and two with dedifferentiated chondrosarcoma) with paclitaxel 175 mg/m$^2$. No responses, other than one mixed response, were observed. Four paediatric patients with osteosarcoma included in two separate phase I studies of paclitaxel in paediatric solid tumours also failed to respond to paclitaxel chemotherapy.[32,33]

Although many of the patients in this study had been heavily pre-treated, the drug was well tolerated with only four episodes of neutropenic sepsis seen. All other toxicities were easily managed in the outpatient setting.

In conclusion, docetaxel at 100 mg/m$^2$ as a 1-h infusion is well tolerated, but is not effective in relapsed or refractory osteosarcoma. Similar results seen in other small studies suggest that taxanes have no role to play in the treatment of osteosarcoma. Further combination studies with platinum compounds do not therefore appear to be warranted.

### References

1. Souhami RL, Craft AW, Van der Eijken JW, Nooij M, Spooner D, Bramwell VH, *et al.* Randomised trial of two regimens of chemotherapy in operable osteosarcoma: a study of the European Osteosarcoma Intergroup. Lancet 1997; 350: 911–7.
2. Smeland S, Muller C, Alvegard TA, Wiklund T, Wiebe T, Bjork O, *et al.* Scandinavian Sarcoma Group Osteosarcoma Study SSG VIII: prognostic factors for outcome and the role of replacement salvage chemotherapy for poor histological responders. *Eur J Cancer* 2003; 39: 488–94.
3. Meyers PA, Gorlick R, Heller G, Casper E, Lane J, Huvos AG, *et al.* Intensification of preoperative chemotherapy for osteogenic sarcoma: results of the Memorial Sloan-Kettering (T12) protocol. *J Clin Oncol* 1998; 16: 2452–8.
4. Fuchs N, Bielack SS, Epler D, Bieling P, Delling G, Korholz D, *et al.* Long-term results of the co-operative German-Austrian-Swiss osteosarcoma study group's protocol COSS-86 of intensive multidrug chemotherapy and surgery for osteosarcoma of the limbs. *Ann Oncol* 1998; 9: 893–9.
5. Bacci G, Briccoli A, Mercuri M, Ferrari S, Bertoni F, Gasbarrini A, *et al.* Osteosarcoma of the extremities with synchronous lung metastases: long-term results in 44 patients treated with neoadjuvant chemotherapy. *J Chemother* 1998; 10: 69–76.
6. Bielack SS, Kempf-Bielack B, Delling G, Exner GU, Flege S, Helmke K, *et al.* Prognostic factors in high-grade osteosarcoma of the extremities or trunk: an analysis of 1,702 patients treated on neoadjuvant cooperative osteosarcoma study group protocols. *J Clin Oncol* 2002; 20: 776–90.
7. Ferrari S, Briccoli A, Mercuri M, Bertoni F, Picci P, Tienghi A, *et al.* Postrelapse survival in osteosarcoma of the extremities: prognostic factors for long-term survival. *J Clin Oncol* 2003; 21: 710–5.
8. Bielack SS, Kempf-Bielack B, Branscheid D, Helmke K, Kabisch H, Kotz R, *et al.* Relapsed osteosarcoma: An analysis of 576 Cooperative Osteosarcoma Study Group (COSS) patients. *Proc Am Soc Clin Oncol* 2003; 22: 3305.
9. Saeter G, Hoie J, Stenwig AE, Johansson AK, Hannisdal E, Solheim OP. Systemic relapse of patients with osteogenic sarcoma. Prognostic factors for long term survival. *Cancer* 1995; 75: 1084–93.
10. Ringel I, Horwitz SB. Studies with RP 56976 (taxotere): a semisynthetic analogue of taxol. *J Natl Cancer Inst* 1991; 83: 288–91.
11. McGuire WP, Rowinsky EK, Rosenshein NB, Grumbine FC, Ettinger DS, Armstrong DK, *et al.* Taxol: a unique antineoplastic agent with significant activity in advanced ovarian epithelial neoplasms. *Ann Intern Med* 1989; 111: 273–9.
12. Gore ME, Levy V, Rustin G, Perren T, Calvert AH, Earl H, *et al.* Paclitaxel (Taxol) in relapsed and refractory ovarian cancer: the UK and Eire experience. *Br J Cancer* 1995; 72: 1016–9.
13. Holmes FA, Walters RS, Theriault RL, Forman AD, Newton LK, Raber MN, *et al.* Phase II trial of taxol, an active drug in the treatment of metastatic breast cancer. *J Natl Cancer Inst* 1991; 83: 1797–805.
14. Chan S, Friedrichs K, Noel D, Pinter T, Van Belle S, Vorobiof D, *et al.* Prospective randomized trial of docetaxel versus doxorubicin in patients with metastatic breast cancer. The 303 Study Group. *J Clin Oncol* 1999; 17: 2341–54.
15. Ferraresi V, Milella M, Vaccaro A, D'Ottavio AM, Papaldo P, Nistico C, *et al.* Toxicity and activity of docetaxel in anthracycline-pretreated breast cancer patients: a phase II study. *Am J Clin Oncol* 2000; 23: 132–9.
16. Sulkes A, Smyth J, Sessa C, Dirix LY, Vermorken JB, Kaye S, *et al.* Docetaxel (Taxotere) in advanced gastric cancer: results of a phase II clinical trial. EORTC Early Clinical Trials Group. *Br J Cancer* 1994; 70: 380–3.
17. Murphy WK, Fossella FV, Winn RJ, Shin DM, Hynes HE, Gross HM, *et al.* Phase II study of taxol in patients with untreated advanced non-small-cell lung cancer. *J Natl Cancer Inst* 1993; 85: 384–8.
18. Meyer T, McTiernan A, Whelan J. A phase II study of docetaxel in patients with relapsed and refractory Ewing's tumours. *Sarcoma* 2003; 7: 13–7.
19. Edmonson JH, Ebbert LP, Nascimento AG, Jung SH, McGaw H, Gerstner JB. Phase II study of docetaxel in advanced soft tissue sarcomas. *Am J Clin Oncol* 1996; 19: 574–6.
20. van Hoesel QG, Verweij J, Catimel G, Clavel M, Kerbrat P, van Oosterom AT, *et al.* Phase II study with docetaxel (Taxotere) in advanced soft tissue sarcomas of the adult. EORTC Soft Tissue and Bone Sarcoma Group. *Ann Oncol* 1994; 5: 539–42.
21. Verweij J, Lee SM, Ruka W, Buesa J, Coleman R, van Hoessel R, *et al.* Randomized phase II study of docetaxel versus doxorubicin in first- and second-line chemotherapy for locally advanced or metastatic soft tissue sarcomas in adults: a study of the european organization for research and treatment of cancer soft tissue and bone sarcoma group. *J Clin Oncol* 2000; 18: 2081–6.
22. Peng L, Wang Z, Wang Q. Docitaxol-induction apoptosis of osteosarcoma. *Zhonghua Zhong Liu Za Zhi* 2001; 23: 190–2.
23. Burns BS, Edin ML, Lester GE, Tuttle HG, Wall ME, Wani MC, *et al.* Selective drug resistant human osteosarcoma cell lines. *Clin Orthop* 2001: 259–67.

24. Pronk LC, Schellens JH, Planting AS, van den Bent MJ, Hilkens PH, van der Burg ME, *et al*. Phase I and pharmacologic study of docetaxel and cisplatin in patients with advanced solid tumors. *J Clin Oncol* 1997; 15: 1071–9.
25. Millward MJ, Zalcberg J, Bishop JF, Webster LK, Zimet A, Rischin D, *et al*. Phase I trial of docetaxel and cisplatin in previously untreated patients with advanced non-small-cell lung cancer. *J Clin Oncol* 1997; 15: 750–8.
26. Fossella F, Pereira JR, von Pawel J, Pluzanska A, Gorbounova V, Kaukel E, *et al*. Randomized, multinational, phase III study of docetaxel plus platinum combinations versus vinorelbine plus cisplatin for advanced non-small-cell lung cancer: the TAX 326 study group. *J Clin Oncol* 2003; 21: 3016–24.
27. World Health Organisation. *Handbook for reporting results of cancer treatment*. World Health Organisation Offset Publication No. 48. Geneva: World Health Organisation, 1979.
28. Cancer Therapy Evaluation Program. Common Toxicity Criteria, Version 2.0. DCTD, NCI, NIH, DHHS March, 1998:
29. Blaney SM, Seibel NL, O'Brien M, Reaman GH, Berg SL, Adamson PC, *et al*. Phase I trial of docetaxel administered as a 1-hour infusion in children with refractory solid tumors: a collaborative pediatric branch, National Cancer Institute and Children's Cancer Group trial. *J Clin Oncol* 1997; 15: 1538–43.
30. Seibel NL, Blaney SM, O'Brien M, Krailo M, Hutchinson R, Mosher RB, *et al*. Phase I trial of docetaxel with filgrastim support in pediatric patients with refractory solid tumors: a collaborative Pediatric Oncology Branch, National Cancer Institute and Children's Cancer Group trial. *Clin Cancer Res* 1999; 5: 733–7.
31. Patel SR, Papadopoulos NE, Plager C, Linke KA, Moseley SH, Spirindonidis CH, *et al*. Phase II study of paclitaxel in patients with previously treated osteosarcoma and its variants. *Cancer* 1996; 78: 741–4.
32. Doz F, Gentet JC, Pein F, Frappaz D, Chastagner P, Moretti S, *et al*. Phase I trial and pharmacological study of a 3-hour paclitaxel infusion in children with refractory solid tumours: a SFOP study. *Br J Cancer* 2001; 84: 604–10.
33. Hayashi RJ, Blaney S, Sullivan J, Weitman S, Vietti T, Bernstein ML. Phase 1 study of Paclitaxel administered twice weekly to children with refractory solid tumors: a pediatric oncology group study. *J Pediatr Hematol Oncol* 2003; 25: 539–42.
